# The Recent Advances in the Mechanical Properties of Self-Standing Two-Dimensional MXene-Based Nanostructures: Deep Insights into the Supercapacitor

**DOI:** 10.3390/nano10101916

**Published:** 2020-09-25

**Authors:** Yassmin Ibrahim, Ahmed Mohamed, Ahmed M. Abdelgawad, Kamel Eid, Aboubakr M. Abdullah, Ahmed Elzatahry

**Affiliations:** 1 Center for Advanced Materials, Qatar University, Doha 2713, Qatar; yi1511403@student.qu.edu.qa; 2 Department of Mechanical and Industrial Engineering, College of Engineering, Qatar University, Doha 2713, Qatar; a.mohamed@qu.edu.qa; 3 Gas Processing Center, College of Engineering, Qatar University, Doha 2713, Qatar; aabdelga14@gmail.com; 4 Materials Science and Technology Program, College of Arts and Sciences, Qatar University, Doha 2713, Qatar

**Keywords:** MXene, mechanical properties, 2D materials, metal carbide, young modules, supercapacitors

## Abstract

MXenes have emerged as promising materials for various mechanical applications due to their outstanding physicochemical merits, multilayered structures, excellent strength, flexibility, and electrical conductivity. Despite the substantial progress achieved in the rational design of MXenes nanostructures, the tutorial reviews on the mechanical properties of self-standing MXenes were not yet reported to our knowledge. Thus, it is essential to provide timely updates of the mechanical properties of MXenes, due to the explosion of publications in this filed. In pursuit of this aim, this review is dedicated to highlighting the recent advances in the rational design of self-standing MXene with unique mechanical properties for various applications. This includes elastic properties, ideal strengths, bending rigidity, adhesion, and sliding resistance theoretically as well as experimentally supported with various representative paradigms. Meanwhile, the mechanical properties of self-standing MXenes were compared with hybrid MXenes and various 2D materials. Then, the utilization of MXenes as supercapacitors for energy storage is also discussed. This review can provide a roadmap for the scientists to tailor the mechanical properties of MXene-based materials for the new generations of energy and sensor devices.

## 1. Introduction

**Scheme 1:**
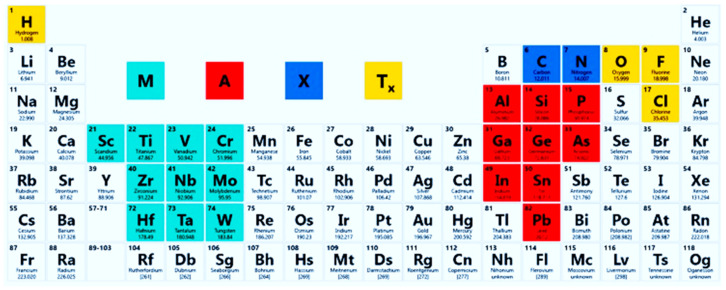
The composition of MXenes and MAX phases from the periodic table.

Carbon-based nanostructures (C-Ns) such as graphene, carbon nanotubes, and carbon nitride are of great interest due to their unique physiochemical merits such as high surface area, thermal stability, and outstanding mechanical properties [1,2,3,4]. These properties promoted the utilization of C-Ns in structural composites, protective coatings, fibers, energy storage, catalysis, and durable wearable sensors; however, their complicated fabrication process remains a major challenge [5,6,7]. Y. Gogotsi and M.W. Barsoum groups discovered a novel family of 2D transition metal carbides or nitrides called MXene (pronounced “maxenes”) [8]. The general formula of MXene is M_n+1_X_n_T_x_ (*n* = 1–4), where M represents transition metals, A is an A-group element of group 13 to 15 in the periodic table, X is carbon or nitrogen, and T_x_ is surface functional groups (OH, O, Cl, F) (Scheme 1) [8]. There are around three main structures of MXenes, including M_2_XT_x_, M_3_X_2_T_x_, and M_4_X_3_T_x,_ derived from the selective etching of MAX phases (M, A, and X elements are in Scheme 1) including M_2_AX, M_3_AX_2_, and M_4_AX_3_. To this end, more than 30 MXenes compositions have prepared, such as Ti_2_CT_x_, Nb_2_CT_x_, V_2_CT_x_, Ti_3_C_2_T_x_, Mo_2_TiC_2_T_x_, Mo_2_Ti_2_C_3_, Ti_y_Nb_2−y_CT_x_, and Nb_y_V_2−y_CT_x_, along with additional dozens were explored by computational methods [9,10,11]. 

MXenes possess unique physical and chemical merits such as great miscibility, high surface area to volume ratio, accessible active sites, surface charge state, electron-rich density, and absorption of electromagnetic waves [12]. This is besides the impressive properties of 2D carbide transition metal carbides/nitrides, such as multilayered structures with excellent mechanical properties, strength, flexibility, and high electrical conductivity [12]. Additionally, the fabrication process of MXene is scalable, productive, controllable, facile, and feasible for large-scale applications [12]. MXenes with high negative zeta potential are miscible in various solvents, polymeric materials, and other C-Ns materials resulting in the formation of unlimited composites with various properties [13]. The impressive mechanical properties of MXenes are one of the unique features for MXene [2,14,15,16]. Despite the significant progress in the synthesis of MXene nanostructures, Ti_3_C_2_T_x_ compound is the most widely studied material, for various applications, due to its impressive electrical conductivity, mechanical properties, and electrochemical properties electromagnetic shielding [2,14,15,16].

There are numerous published reviews in the fields of MXenes for energy, catalysis, and environmental remediation [12,17,18,19,20,21,22,23]. However, the reviews on the mechanical properties of self-standing MXenes are not yet reported [24]. Many studies have shown that MXenes exhibits excellent mechanical ion adsorption properties, which in turn will set the stage for exploring the possibility of their use in sensors and flexible devices [6,24,25,26]. For instance, the strain-tunable electrochemical properties of MXenes enable them to be a propitious solution for flexible and stretchable devices [6,24,25,26]. Regarding the electrochemical properties of MXenes, their large specific surface area makes them a promising candidate for various applications such as supercapacitor, Li-ion and Sodium-ion batteries, hydrogen storage, adsorption, and catalysts [6,24,25,26]. Due to the abundant research and ceaseless publications on the mechanical properties of MXene (more than 146 articles, according to SciFinder), it is crucial to provide a timely update of research efforts in this area.

Inspired by this, the presented review summarizes the recent progress of research work on the mechanical properties of self-standing MXenes, from both theoretical and experimental views. This includes: (1) elastic properties and superior strengths, (2) bending rigidity, (3) adhesion, and sliding resistance with their fundamental mechanism supported with numerous representative paradigms. Also, there are deep insights into the utilization of MXenes as supercapacitors. The future perspective of the mechanical applications of MXene is also discussed.

## 2. Mechanical Properties of Self-Standing MXenes

In this section, the elastic properties and superior strengths of self-standing MXenes are briefly summarized, and we discuss the effect of other parameters such as layer thickness, functional groups, and presence of point defects, different transition metals, and substitutional doping. The mechanical properties of MXenes with different compositions are summarized in Table 1.

### 2.1. Elastic Properties and Ideal Strengths

#### 2.1.1. Effect of Functional Terminations

**Figure 1:**
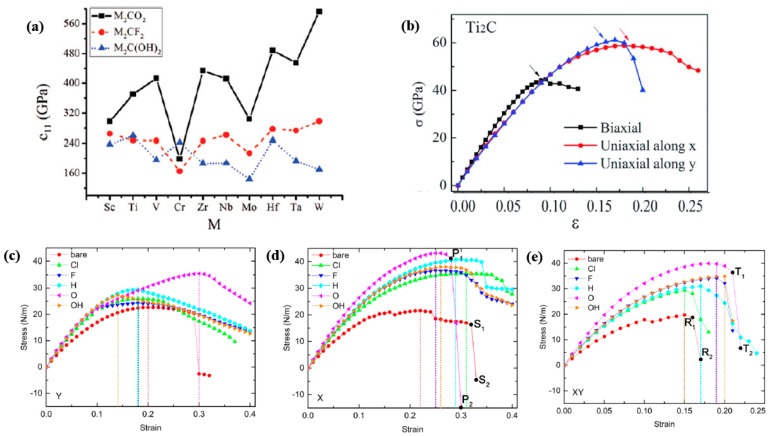
(**a**) The elastic constants of c11 for M_2_CT_2_ MXenes. Reproduced with permission from [27]. Copyright IOP Publishing, 2015. (**b**) Calculated stress-strain curves of 2D Ti_2_C. Reproduced with permission from [37]. Copyright RSC, 2015 (**c**) The stress-strain curves in the uniaxial tension Y direction, (**d**) The stress-strain curves in the uniaxial tension X direction for Ti_3_C_2_ and P_1_ and P_2_ for Ti_3_C_2_O_2_, (**e**) The stress-strain curves in biaxial tension for Ti_3_C_2_ and T_1_ and T_2_ for Ti_3_C_2_O_2_. All the vertical lines mention the maximum stress values. Reproduced with permission from [39]. Copyright APS, 2016.

Functional terminations (–O, –F, –OH) of carbides have a significant effect on the structural and mechanical properties of MXenes, as demonstrated extensively by DFT calculation. Figure 1a shows the variation of the calculated elastic constants c_11_ of M_2_CT_2_ MXenes as a function of the layer thickness and different functional terminations [27]. It can be seen that, except for Cr_2_CO_2_, the elastic constant for MXenes with oxygen functionalization showed higher elastic constants compared to those with hydroxyl and fluorine functional groups [27]. This is due to the stronger interaction between the oxygen and surface M atoms [27].

The stress-strain curves, as well as the deformation mechanisms, were investigated in response to tensile stress by DFT calculation for 2D Ti_n+1_C_n_ (*n* = 1–3) (Figure 1b) [37]. Three loading conditions were considered to measure the intrinsic mechanical responses to tensile strain in 2D Ti_2_C, which are biaxial tension, uniaxial tension along the x-direction, and the y-direction [37]. The stress-strain relations for 2D Ti_2_C under different loading conditions are shown in (Figure 1b) [37]. It was found that 2D Ti_2_C is an elastically isotropic material, since the corresponding Young’s modulus E_x_ and E_y_ were estimated to be 620 GPa and 600 GPa, respectively [37]. Moreover, 2D Ti_2_CO_2_ can sustain higher strains for the three loading conditions than 2D Ti_2_C, which is even higher than that of graphene due to surface functionalizing oxygen [37]. Another large variation in mechanical properties was detected when different transition metal, along with surface functional groups, are used [28,38]. Furthermore, in comparison to other functional groups in Ti_3_C_2_, the oxygen group possesses the highest in-plane planar elastic modulus, as shown in (Figure 1c–e), leading to enhancement of strength, and adsorption energy, which indicates its good thermodynamic stabilization [39]. This can be attributed to the significant charge transfer from inner to outer surface bonds [39].

The effect of surface termination groups on the elastic constants of 2D Ti_2_CT_2_ and Ti_3_C_2_T_2_ was investigated using first-principles calculation by Density-functional theory (DFT) simulation [40]. It was found that the stiffness is highly dependent on the termination group. The elastic stiffness of the MXenes is only maintained in the case of MXenes with O terminations while deteriorates in the case of F and OH terminations [40]. This can be explained by the in-plane lattice constant in the 2D MXenes with different termination groups. The in-plane lattice constant for both 2D Ti_2_CT_2_ and Ti_3_C_2_T_2_ MXenes was shortest in the case of O termination. In contrast, F and OH termination had larger in-plane lattice constants indicating a strong interaction between Ti and terminating O atoms. 

**Figure 2:**
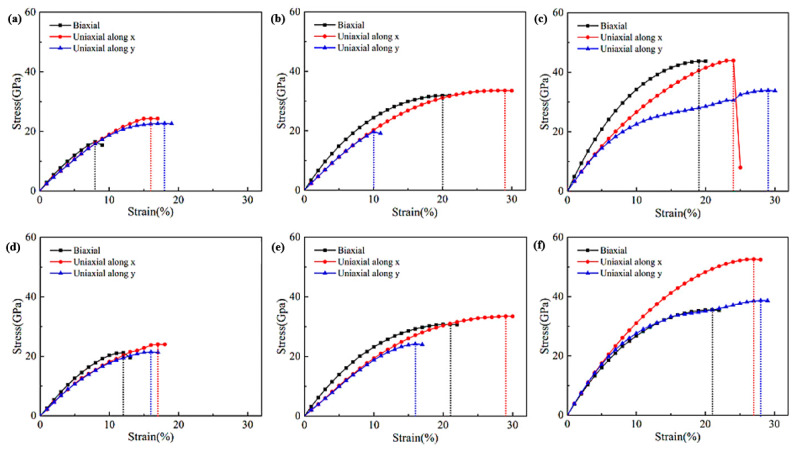
Strain-stress relationships for (**a**) Ti_2_C, (**b**) Ti_2_CF_2_, (**c**) Ti_2_CO_2_, (**d**) Zr_2_C, (**e**) Zr_2_CF_2_, and (**f**) Zr_2_CO_2_ under both biaxial and uniaxial load conditions. Reproduced with permission from [30]. Copyright PNAS, 2017.

**Figure 3:**
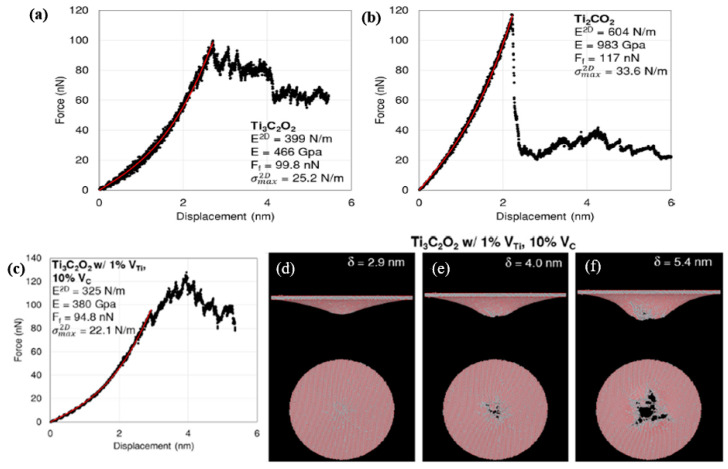
Force-Displacement curves for pristine monolayers of Ti_3_C_2_O_2_ (**a**) and Ti_2_CO_2_ (**b**). (**c**) A representative force-displacement curve. (**d**–**f**) photographs are showing the progressive indentation and fracture of the same representative Ti_3_C_2_O_2_ monolayer with 1% V_Ti_ and 10% V_C_. Reproduced with permission from [32] Copyright Elsevier, 2019.

Another work in the literature [30] studied the effect of surface groups on the elastic properties of MXenes. The ionic mobility for MXenes with different termination groups was investigated under different strain conditions, using multiaxial loading schemes, biaxial and uniaxial tension along x-direction and y-direction. It was observed that Ti_2_C (Ti_2_CF_2_) {Ti_2_CO_2_} can tolerate percentage of strains of 8 (20) {19}, 16 (29) {24}, and 18 (10) {29} under biaxial and uniaxial tensions along the x and y directions, respectively. Whereas Zr_2_C (Zr_2_CF_2_) {Zr_2_CO_2_} can withstand strains of 12 (21) {21}%, 16 (29) {27}% and 17 (16) {28}%, respectively as shown in Figure 2 [30]. It can be seen that Ti_2_CO_2_ has higher critical strains than both Ti_2_C and graphene [30]. Additionally, overall, the surface groups (O and F) increase the critical strain and provide more mechanical flexibility to the 2D MXenes by considerably slowing down the collapse of the transition metal layers. This makes MXenes with O and F termination groups potential candidates for high-performance lithium-ion batteries [30]. A recent study [32] investigated the effect of point defects on the elastic properties of MXenes using the atomistic simulation of nanoindentation of Ti_n+1_C_n_O_2_ monolayer. The Young’s modulus of Ti_3_C_2_O_2_ was found to be 466 GPa, which is slightly lower than the obtained values by DFT and hybridized computational molecular dynamics (MD) simulations of 523 [41] and 502 [42] GPa, respectively. This can be attributed to the presence of surface terminations. Moreover, the breaking strength of Ti_3_C_2_O_2_ was calculated as 25.2 N/m, lower than that of graphene (42 N/m) [43]. As seen in (Figure 3a,b), Ti_2_CO_2_ exhibited a more sudden fracture compared to Ti_3_C_2_O_2_, at higher force and lower displacement [32]. This can be explained by the presence of two fewer atomic layers in Ti_2_CO_2_, resulting in a decreased resistance and more abrupt failure. The calculated elastic modulus of Ti_2_CO_2_ (983 GPa) is higher than that of Ti_3_C_2_O_2_ and almost approaching the value of graphene [32]. However, this value is inconsistent with the previously reported values by DFT (636 GPa) [41] and hybridized MD (597 GPa) [42]. The calculated breaking force for Ti_2_CO_2_ of 33.6 N/m is approaching the levels of graphene [32].

Figure 3c–f shows simulation results of the nanoindentation of Ti_3_C_2_O_2_ with titanium and carbon vacancies (V_Ti_ and V_C,_ respectively) [32]. It can be seen that the cracks failed to propagate to the edges of the samples contained 1% V_Ti_ and 10% V_C_ with the same extent of the pristine Ti_3_C_2_O_2_, which explains the effect of defects on the fracture mechanism of the sheets [32]. Furthermore, the presence of defects results in a 17% reduction in elastic modulus (386 GPa), which is still higher than graphene oxide and in good agreement with the recent experimental studies [2].

#### 2.1.2. Effect of the Mass of the Transition Metal

DFT calculation using the Vienna ab initio simulation package (VASP) code, the mechanical and dynamical properties were obtained for both pristine and terminated MXene (M_2_XT_2_) structures with M = Sc, Mo, Ti, Zr, Hf, X = C, N, and T = O, F [29]. It was found that for the pristine carbides, unlike nitride-based pristine, there is a positive correlation between the stiffness and the mass of the transition metal, as indicated by elastic constants [29]. Moreover, the Young Modulus for the nitrides was slightly higher than that of the carbides [29].

In a recent study [44], the effect of asymmetrical functionalization of F and OH groups on the mechanical properties of monolayer Janus MXenes M_2_X (M = Sc, Ti, V, Mn, Nb, Mo, Hf; X = C, N) where the X atomic layer is sandwiched between 2 M layers was studied via DFT. It was found that mechanical properties depend on the mass of the transition metal and the surface functionalization. Results show that asymmetric functionalization has a consequential effect on the elastic properties of the MXenes. For all the pristine M_2_X, the in-plane stiffness C of M_2_C is slightly lower than that of M_2_N due to the additional valence electron that the N atom provides than C atoms than in turn generate stiffer M-X bonds. However, due to the H structure of Mo_2_X, the in-plane stiffness of Mo_2_N is slightly lower than that of Mo_2_C. Another finding was that by asymmetrical F/OH surface functionalization, the in-plane stiffness C of Sc_2_C was increased from 92 Nm^−1^ to 192 Nm^−1^, which agrees with what was found by [45]. Moreover, the in-plane stiffness C of monolayer M_2_X is lower than that of both graphene [46] and single layer h-BN [47]. Upon asymmetrical surface functionalization, the mechanical stability and the in-plane stiffness C of monolayer M_2_X can be enhanced [44]. Despite the fact that it is experimentally challenging to synthesize MXenes accompanied with mixed functional groups [48], eventually, the Janus MXenes could be synthesized experimentally, similar to the Janus graphene [33] and Janus graphene oxide [49].

#### 2.1.3. New Types of MXenes

**Figure 4:**
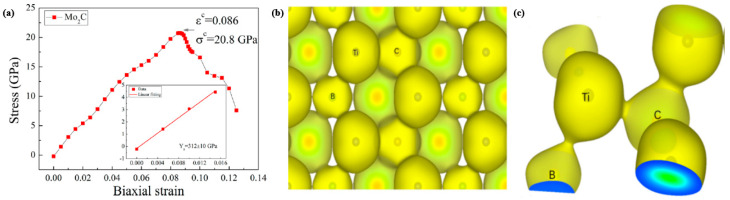
(**a**) Calculated stress versus biaxial strain for the Mo_2_C. Reproduced with permission from [34]. Copyright ACS Publications, 2016. (**b**) Shows the plot in an extended region, (**c**) zoomed view focused on central Ti atom bonded with B and C. The stronger covalency of the Ti-C bond compared to the Ti-B bond is visible. Reproduced with permission from [51]. Copyright AIP Publishing, 2017.

The enhanced mechanical properties of new types of MXenes, such as Mo_2_C were predicted by DFT calculations [34]. The Mo_2_C was fabricated via the chemical vapor deposition (CVD) method, where the carbon source was methane, and Cu-foil was selected to be the substrate for a molybdenum foil [50]. The lateral size of the fabricated Mo_2_C was found to be >100 µm [50]. No significant structural changes were observed after immersing Mo_2_C in several solvents such as isopropanol, ethanol, HCl, or after thermal annealing in air at 200 °C for 2 h, indicating its thermal and chemical stability [50]. Compared to the MoS_2_, Mo_2_C had a slightly higher biaxial elastic modulus of 312 ± 10 GPa [34]. The relatively large elastic modulus could be explained by the strong interactions between Mo and C atoms. The calculated stress-strain curve for Mo_2_C (Figure 4a) shows mostly an elastic response until a critical strain of 0.086, then the Mo_2_C exhibited creep deformation. Although this critical strain is less than that of MoS_2_, the ideal strength of Mo_2_C is predicted as 20.8 GPa, approaching the value of a monolayer of MoS_2_ (23.8) GPa [34]. The impressive mechanical properties make Mo_2_C a potential candidate for mechanical applications.

#### 2.1.4. Effect of Doping

**Figure 5:**
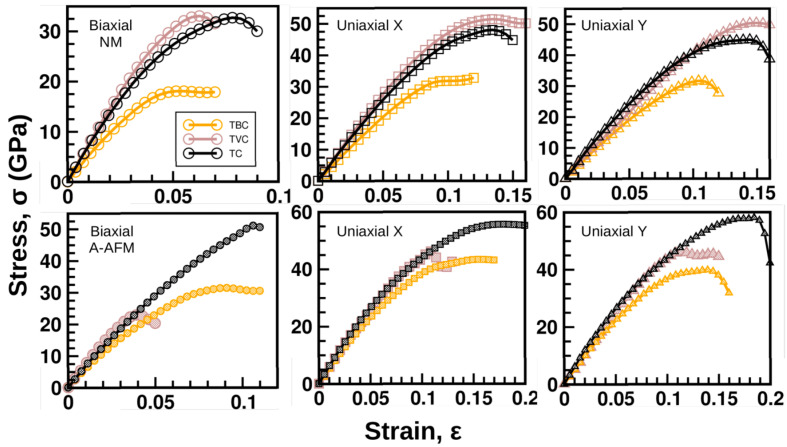
The stress-strain curve of Ti_2_C, Ti_2_(C_0.5_B_0.5_) and (Ti, V)C under biaxial and uniaxial tensile strains along with the X and Y directions. The top panels show the results for NM states, while the bottom panels show the results for the minimum energy AFM magnetic state. Reproduced with permission from [51]. Copyright AIP Publishing, 2017.

The effect of doping on the elastic properties of MXenes was investigated by DFT calculations [51]. Specifically, B and V atoms were substitutionally doped into Ti and C sites in Ti_2_C, respectively, resulting in Ti_2_(C_0.5_B_0.5_) and (Ti, V)C. While V-doping results only in marginal enhancement, B-doping yields improved the elastic properties by decreasing the in-plane Young’s modulus and the yield strength. The reduction in the stiffness can be attributed to the weak-bond of Ti-B compared to the Ti-C bond (Figure 4b,c) [51]. Figure 5 shows the calculated stress-strain curves using the non-magnetic (NM) and the lowest energy antiferromagnetic (AFM) states [51]. It can be seen that a remarkable decrease of about 25–27% in Young’s modulus and in-plane stiffness of Ti_2_(C_0.5_B_0.5_) compared to Ti_2_C. However, the doping of V at Ti sites results in the same stiffness of the undoped Ti_2_C. Intriguingly, the stiffness of Ti_2_(C_0.5_B_0.5_) was about 4.2, 1.5, 1.86, and 3.1 times higher than that of 2D MoS_2,_ graphene, *h*-BN, and SiC reported elsewhere, respectively, due to the B-doping effect [51,52]. In contrast, Ti_2_(C_0.5_B_0.5_) and (Ti_,_V)C with O-termination groups exhibited improved elastic properties compared to undoped Ti_2_C O-passivated or O-free, owing to the enhancement of the local strain, causing a consequent enlarging of the average thickness of O-passivated MXene by nearly 2% [51].

**Figure 6:**
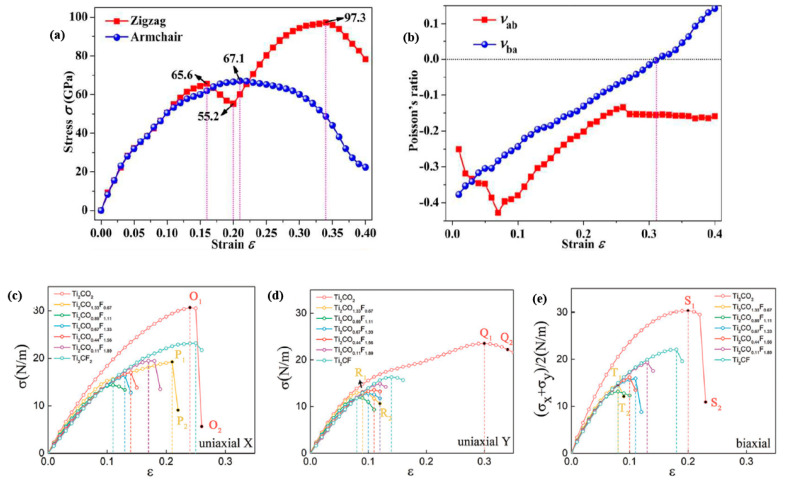
(**a**) Stress-strain curves of intrinsic W_2_C under uniaxial stretching, (**b**) Poisson’s ratios of W_2_C stretched along with different directions. Reproduced with permission from [53]. Copyright RSC, 2018. The calculated stress σ versus strain ε curves for Ti_2_CT_2_ with pure and mixed terminations under (**c**) uniaxial *x*, (**d**) uniaxial *y*, and (**e**) biaxial loading conditions. Reproduced with permission from [55]. Copyright John Wiley & Sons, 2018.

Another study [53] predicted enhanced elastic properties of a 2D Tungsten Carbide (W_2_C) monolayer by DFT calculations. The calculated c_11_ of 781.9 GPa indicated that W_2_C is mechanically stable as it satisfies the 2D materials criteria for mechanical stability [36]. The uniaxial tensile loading was applied along the armchair direction, where the correlation between the strain and stress was investigated. As the strain increases, the stress increases until approaching the ultimate tensile strength, as shown in (Figure 6a), then it decreases gradually. The same trend goes for the calculations along the zigzag direction. The calculated ultimate strength of W_2_C is comparable to Ti_2_C [37] but higher than that of MoS_2_ [54]. The Young’s modulus of W_2_C along with the armchair and zigzag directions are 648 and 645 GPa, respectively, compared to graphene (1000 GPa) [43] and Ti_2_C (600 GPa) [37]. Furthermore, W_2_C was observed to have a high negative Poisson’s ratio (NPR) as they exhibited a positive strain along the longitudinal direction while applying stretching force on the transverse direction (Figure 6b) [53]. This intrinsic NPR of W_2_C could be explained by the robust coupling between C-p and W-d orbitals in the pyramid structural unit. Additionally, incorporating the surface functional groups to the calculations show that the NPR of W_2_C was turned into PPR due to the weakening of M–C interactions [53].

#### 2.1.5. Effect of Varying F/O Ratio

The mechanical properties of Ti_2_CT_n_ terminated by O− and F− were manipulated by varying F/O ratio from 1:17 to 17:1 [55]. The mechanical properties of the five thermodynamically most stable structures with F/O ratios 1: 2, 5:4, 2:1, 7:2, and 17:1 were investigated. It was noticed that as the F/O ratio increases, the Young’s, along the x- and y-directions, and shear moduli of Ti_2_CT_2_ gradually decrease. For instance, Young’s modulus along the x-direction decreased from 222 to 159 (N m^−1^), while the shear modulus decreased from 88 to 58 (N m^−1^). Additionally, all the Ti_2_CT_2_, with pure and a mixture of surface terminations, exhibited Poisson ratios greater than that of graphene (0.224), ranging from 0.24 to 0.37. Ti_2_CO_1.33_F_0.67_ exhibits the highest Young’s modulus along the x-direction (222 N m^−1^), among other Ti_2_CT_2_, which is comparable to Ti_2_CO_2_ (241 N m^−1^). The calculated stress-strain curves shown in (Figure 6c–e) indicated that Ti_2_CO_2_ (24%, 30%, and 20% under uniaxial tensions along the *x*- and *y*-directions and under biaxial tensions) and Ti_2_CF_2_ (25%, 14%, and 18%) have higher critical strains than that of Ti_2_CT_2_ with mixed terminations. Thus, the mechanical flexibility could be decreased with mixed functional groups. Moreover, a lower critical strain of Ti_2_CT_2_ is observed with a higher degree of the mixture (Figure 6c–e), such as in Ti_2_CO_0.11_F_1.89_, which could sustain only the lowest critical stains. A similar trend is seen for the ideal strength with the variation of the degree of mixture, indicating that the best mechanical strengths are assigned to Ti_2_CO_1.33_F_0.67_ and Ti_2_CO_0.11_F_1.89_. These findings pave the way to demonstrate more effective methods in tuning the electrochemical properties of MXenes by strains.

#### 2.1.6. Effect of Number of Layers and Layer Thickness

The elastic modulus and breaking strength of monolayer and bilayer Ti_3_C_2_T_x_ flakes were experimentally determined by AFM indentation [2]. It was shown that E^2D^ values determined for bilayer Ti_3_C_2_T_x_ flakes are exactly twice that determined for monolayer MXene membranes, suggesting strong interaction between the layers due to hydrogen bonding. A single layer of Ti_3_C_2_T_x_ has an effective Young’s modulus of approximately 333 GPa, which is higher than that of graphene oxide (210 GPa) and some other MXenes. Meanwhile, the breaking strength of a single layer of Ti_3_C_2_T_x_ was 17.3 ± 1.6 GPa. It was noted that Young’s modulus obtained experimentally is lower than that from the MD simulation due to the presence of defects and surface functionalization.

The effect of layer thickness on the structural and elastic properties of 2D Ti_n+1_C_n_ was studied using MD calculations [42]. It was demonstrated that the Young’s modulus of MXenes could be significantly increased by decreasing the layer thickness. The Young’s modulus of Ti_2_C, Ti_3_C_2,_ and Ti_4_C_3_ was found to be 597, 502, and 534 GPa, respectively, with a strain ε less than 0.01 and within 10% interpolation error. As observed, the highest Young’s modulus was reported to the thinnest Ti_2_C carbide (3 atomic layers). These results are in agreement with other theoretical predictions from DFT [41]. 

Similar findings on the effect of monolayer thickness on the elastic properties of the carbide (Ti_n+1_C_n_) and nitride-based (Ti_n+1_N_n_) MXenes, by DFT calculations, were reported in another study [53]. It was shown that increasing the monolayer thickness decreases Young’s moduli of MXenes. The Young’s moduli of the Ti_n+1_C_n_ were found to be 601, 473, and 459 GPa for Ti_2_C, Ti_3_C_2_, and Ti_4_C_3_, respectively, which is in good agreement with the values obtained previously by DFT calculations [37]. The bulk model was generated by increasing the layer of atoms to infinity, thus showing that bulk Ti_n+1_C_n_ has Young’s modulus of 433 GPa, lower than that of rest of Ti_n+1_C_n_ MXenes. Although a similar trend was observed for the Ti_n+1_N_n_, higher Young’s moduli of Ti_n+1_N_n_ over Ti_n+1_C_n_ was observed, which is consistent with previously reported experimental measurements for bulk TiN [56] and bulk TiC [57,58]. Furthermore, due to the 2D morphology of Ti_n+1_C_n_ and Ti_n+1_N_n_ with lower thickness, their calculated in-plane Poisson’s ratios (ν) are 0.25 and 0.26, higher than that of the bulk TiC and TiN (~0.23) [56,57,58], which is indicative of increased elasticity.

#### 2.1.7. Effect of Intercalated Ions and Electrolytes

**Figure 7:**
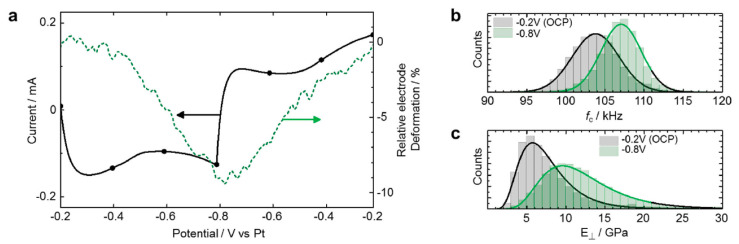
Elastic changes of Ti_3_C_2_T_x_ in Li_2_SO_4_ electrolyte. (**a**) Electrochemical profile of Li^+^ intercalation/extraction showing the current and single point relative electrode deformation profiles as a function of potential. (**b**) Frequency distribution histograms at charged and discharged states. (**c**) Corresponding elastic modulus distribution histograms. Reproduced with permission from [59]. Copyright John Wiley & Sons, 2016.

In order to study the effect of the intercalated ions on the mechanical properties of MXenes, the mechanical properties were characterized at the nanoscale instead of at the macroscopic scale [59]. The elastic changes of a 2D Ti_3_C_2_T_x_ based electrode in a direction normal to the basal plane were studied via in-situ contact resonance force microscopy (CRFM) imaging, combined with DFT during alkaline cation intercalation/extraction [59]. The DFT calculations agreed well with experiments since the presence of only 12.5% H_2_O resulted in a drastic decrease of E from 126 GPa of the dry sample to 29 GPa. The out-of-plane elastic modulus significantly correlated with the cations content. The MXene electrode exhibited shrinkage of almost 10% (Figure 7a) in its lattice structure associated with a decrease in the interlayer distance after Li^+^ intercalation [59].

The Ti_3_C_2_T_x_ exhibited smaller volume changes when K^+^ ions were intercalated, resulting in lower stiffness than in the case of Li^+^ ions [59]. This is possible because the stiffness of the cation/water/MXene system is enhanced by the strong oxygen atoms bonds resulted from one hydrogen atom, from the surface hydroxyl group, being pushed out by the cations. Higher CR frequency values after Li^+^ intercalation indicates a stiffer 2D structure, in the direction normal to the electrode surface, with elastic moduli ranging between 5 and 18 GPa (Figure 7b,c), twice that of water [59]. Additionally, it was found that the elastic modulus can be tuned using the right combination of the electrolyte and the electrode. The use of both the CRFM technique and DFT calculations revealed that the interface between the electrode/electrolyte could be controlled by probing the mechanical properties associated with the cation storage for applications such as supercapacitors and various types of batteries [59].

### 2.2. Bending Rigidity

The mechanical response of 2D materials obtained under bending deformations is a critical quantity called the bending rigidity [24]. The bending rigidity of MXenes is poorly investigated. To the best of our knowledge, only two papers discussing this quantity have been published so far. The bending rigidity is also affected by some parameters, such as the layer thickness and the functionalization group. The bending rigidity of MXenes was first quantified in 2018 using classical MD simulation for three different 2D titanium carbides (Ti_2_C, Ti_3_C_2,_ and Ti_4_C_3_) to demonstrate their bending resistance under applied bending load [60]. Ti_2_C was found to possess higher resistance for bending than atomically thin graphene due to its larger thickness. In contrast, the bending strength of Ti_2_C is lower than that of MoS_2_ due to different atomic arrangements and larger thickness in MoS_2_ compared to Ti_2_C [60].

**Figure 8:**
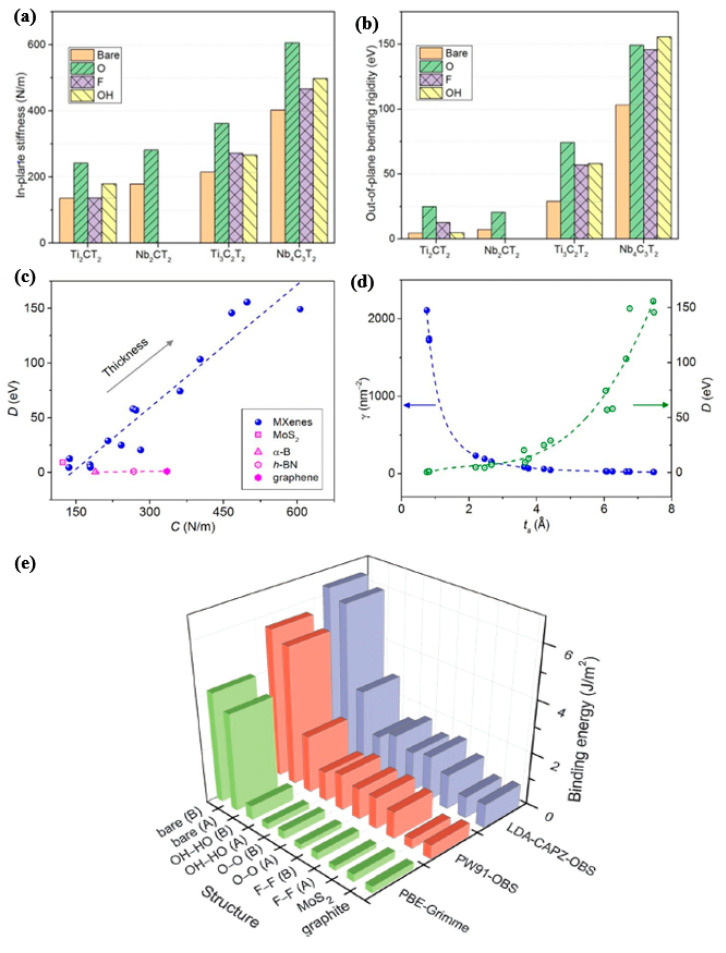
(**a**) In-plane stiffness and (**b**) out-of-plane bending rigidity MXenes. (**c**) Rigidity “D” vs. stiffness, (**d**) *D* vs. *thickness t_s_*. Reproduced with permission from [61]. Copyright RSC, 2020. (**e**) Comparison of the theoretical binding energies of Ti_3_C_2_T_2_, T = OH, F, and O relative to different 2D materials. Reproduced with permission from [65]. Copyright RSC, 2016.

DFT calculations have shown that the in-plane stiffness (C) and out-of-plane bending rigidity (D) of Ti_2_CT_x_, Ti_3_C_2_T_x_, Nb_2_CT_x,_ and Nb_4_C_3_T_x_ (T = O, OH, and F) are highly dependent on the layer thickness of [M_n+1_X_n_] and functionalization groups [61]. As the [M_n+1_X_n_] layer thickness increases, the in-plane stiffness increases (Figure 8a) due to the increase in the number of M-C bonds [61]. Nb_2_CT_2_ and Ti_2_CT_2_ have relatively low in-plane stiffness due to having only a three-atomic layer in [M_2_X], compared to seven-atomic-thick [Nb_4_C_3_] layer in Nb_4_C_3_T_2_ with the largest in-plane stiffness. Moreover, the surface terminations in MXenes significantly increased the stiffness (Figure 8a) [61]. The O-functionalized MXenes were found to have higher in-plane stiffness than that of bare MXenes due to the strong O−M bonding. However, similar in-plane stiffness was noticed for OH, and F terminated Mxenes [61]. Figure 8b depicts the bending rigidities of the four MXenes and their functional groups, showing that Ti_2_C has a D value of 4.47 eV [61]. Compared to MXenes, the surface terminations groups decreased their stiffness of graphene and graphene oxides [62,63]. Additionally, the measured bending rigidities of Ti_2_C with surface functionalities (4.47 eV) were relatively lower than the previously reported value by MD calculations (5.21 eV) [61], but was higher than that of a graphene monolayer (1.2 eV) [64]. Meanwhile, three-atom-thick Ti_2_C and Nb_2_C revealed superior flexibility (observed by Foppl-von Karman number per unit area γ) and higher in-plane stiffness, compared to three-atom-thick MoS_2_ (9.14 eV) [61]. Therefore, increasing the layer thickness decreases the flexibility of MXenes; however, better flexibility could be observed in MXenes with OH terminations, and the thinnest MXenes with a noticeable decrease in the in-plane stiffness (requires milder exfoliation techniques) [61]. As observed in Figure 8c, with increasing the layer thickness of MXenes, the in-plane stiffness, and out-of-plane bending rigidity increases [61]. Lastly, the bending rigidity increases with effective thickness t_s_ in a cubic manner, as presented in Figure 8d where C/D ratios were plotted and γ and D considered as a function of effective thickness for 2D materials [61].

### 2.3. Interlayer Adhesion and Sliding

The interlayer adhesion energy and sliding resistance are two critical characteristics of MXenes that are affected by several parameters such as composition, shape, adhesion, and functional species. For instance, understanding the adhesion between MXenes and various substrates is crucial for MXene device fabrication and performance. DFT calculations demonstrated that the surface functionalities (T = OH, F, and O) weaken interlayer coupling of Ti_3_C_2_T_2_, relative to the bare counterparts as well as other different 2D materials (Figure 8e) [65]. The binding energies of stacked Ti_n+1_C_n_T_2_ were found to be about 2- to 6-fold those of 2D graphite and MoS_2_ materials with weak interlayer coupling. The interlayer coupling of Ti_3_C_2_T_2_ depends on the surface functionalities, which decrease the interlayer coupling, resulting in exfoliation of the stacked Ti_3_C_2_T_2_ into monolayers with outstanding mechanical properties compared to other 2D materials. The OH-containing functionalities were the most strongly coupled Ti_3_C_2_T_2_ with the highest mechanical properties. The determined Young’s moduli normal to the layer plane was 226 GPa for Bernal-Ti_3_C_2_(OH)_2_, which is more energetically preferred and also higher than that of highly oriented pyrolytic graphite (about 34 GPa). Furthermore, another study investigated the effect of surface functionalization on the sliding resistance of M_2_CO_2_ compared to bare counterparts using DFT, along with exploring the strain effect on the sliding resistance [66]. At equilibrium, the layers can easily slide due to the smaller binding energy as a consequence of larger interlayer distance. Due to the oxygen hollow at the surface of oxygen functionalized MXenes, the sliding resistance is increased. However, due to the strong metallic interactions between the stacked M_2_C layers, the sliding resistance is much higher than that of M_2_CO_2_. Another finding is that the relation between the gap and the energy barrier is not linear, whereas as the strain increases, the gap first starts increasing until it reaches maximum value then starts decreasing again. Comparing different stacking configurations, the mirror stacked M_2_CO_2_-II possesses a better lubricant property than the parallel stacked M_2_CO_2_-I because its sliding energy barrier is much lower. In addition, the sliding barrier can be significantly enhanced by normal compression. Whereas, the interlayer sliding, owing to the transfer of different charges from M to O atom, may effectively be hindered by the in-plane biaxial tension. The minimum energy pathway can be modified entirely by the uniaxial tension strain due to anisotropic expansion of the surface electronic state. The functionalized MXenes with strain-controllable frictional properties promise lubricating materials due to their lower sliding resistance and superior mechanical properties.

Another study [67] investigated the effect of point defects on the friction coefficients using DFT calculations and classical MD simulations with reactive force-field (ReaxFF) potentials. The results revealed that the sliding pathways are with low energy barriers in all Ti_n+1_C_n_ (*n* = 1, 2, and 3) systems. For these systems, both DFT and ReaxFF methods predicted friction coefficients for interlayer sliding, for normal loads below 1.2 GPa, to be between 0.24 and 0.273. It was found that titanium (Ti) vacancies in sublayers and terminal oxygen (O) vacancies at surfaces increased the friction coefficients, reaching almost 0.31. That is because the surface roughness increased, resulting in additional attractive forces between adjacent layers. Thereby, Ti_3_C_2_ with surfaces functionalized with –OH and –OCH_3_ groups were studied and found to be able to reduce the friction coefficient to 0.10 and 0.14, respectively.

Understanding of the adhesion among MXenes and different substrates is crucial for the fabrication of MXene devices. In this regard, the adhesion of Ti_3_C_2_T_x_ and Ti_2_CT_x_) with a SiO_2_-coated spherical Si tip was benchmarked compared to graphene (mono-, bi-, and tri-layer) and SiO_2_-coated Si tip substrate using direct AFM measurements [68]. This is based on using the Maugis-Dugdale theory for conversion of the adhesion force measured by the AFM to adhesion energy with consideration of the surface roughness [68]. The average adhesion energies of Ti_3_C_2_T_x_ (0.90  ±  0.03 J m^−2^) was higher than that of Ti_2_CT_x_ (0.40  ±  0.02 J m^−2^) and was in the range of adhesion between graphene and SiO_2_. The superior adhesion energy between SiO_2_ and Ti_3_C_2_T_x_ is due to its thicker monolayer relative to Ti_2_CT_x_. Another observation was that the adhesion energy of multilayer MXene stacks is dependent on the number of monolayers, in contrast to graphene, which is attributed to the larger interlayer spacing and monolayer thickness of the MXenes.

## 3. Self-Standing MXene as Electrode for Supercapacitors

Supercapacitors are highly efficient energy storage devices, owing to their excellent power density, fast charge propagation, and long-term durability. The capacitance performance can be calculated using the maximum stored energy (E) and this equation E = ½ CV^2^, where C is the total capacitance, and V is the working voltage. Meanwhile, the power delivery (P) can be calculated using this equation P = V^2^/4R, where R is the equivalent series resistance of the supercapacitor. Self-standing MXenes are among the most promising materials for supercapacitors due to their excellent electrical conductivity, mechanical flexibility, high surface area, and high capacitance [24,69,70]. Thereby, few reviews emphasized the utilization of MXenes as supercapacitors, which showed that self-standing Ti_3_C_2_T_x_ is the most studied MXenes [71,72,73]. Several factors determine the capacitance performance of MXenes, such as their morphology, surface area, composition, preparation approaches, as well as the type of electrolytes. Table 2 shows the utilization of self-standing Ti_3_C_2_T_x_ prepared by various approaches as efficient supercapacitors, which showed comparable or better performance than that of hybrid Ti_3_C_2_T_x_ (i.e., combined with PPy, rGO, and CNTs) [74,75,76]. Table 3 shows the supercapacitance performance of self-standing Ti_3_C_2_T_x_ and hybrid Ti_3_C_2_T_x_ in different electrolytes solutions. The performance of both self-standing and hybrid Ti_3_C_2_T_x_ in acidic electrolytes (H_2_SO_4_) was significantly higher than that in alkaline or neutral electrolytes (Table 3). For example, Ti_3_C_2_T_x_ showed capacitance performance of 70, 95, 245, and 450 Fg^−1^ in KOH, MgSO_4_, 1 M H_2_SO_4_, and 3 M H_2_SO_4_, respectively [76,77,78]. The same phenomenon was observed in self-standing V_2_CT_x_, which showed the capacitance performance of (487 F g^−1^) in H_2_SO_4_ compared to 225 Fg^−1^ in MgSO_4_ and 184 Fg^−1^ in KOH [79]. Interestingly, the capacitance of self-standing V_2_CT_x_ in H_2_SO_4_ electrolyte (487 F g^−1^) [79] was superior to Ti_3_C_2_T_x_, Mo2CT_x_, Mo_1.33_CT_x_ 245, 196, and 339 F g^−1^, respectively [74,75,80]. The superior capacitances performance of self-standing MXenes in acidic electrolytes compared to in neutral or alkaline electrolytes is owing to the pseudocapacitive performance with surface redox reactions in acidic electrolytes relative to compared to the ion-intercalation capacitance in neutral and alkaline electrolytes [24]. The surface functionalities (i.e., O_2_, OH, and F) have a significant effect on the capacity of the H_2_SO_4_ electrolyte; The increase of O and decrease of F ions termination in Ti_3_C_2_T_x_ increases the capacitance [81].

The pseudocapacitance characteristics and internal mechanism of various MXenes as supercapacitors deeply studied in H_2_SO_4_ electrolyte, in addition to the factors determine the capacitance effect via DFT calculations [81]. This is included various MXenes (M_n+1_X_n_T_x_), where M = Sc, Ti, V, Zr, Nb, Mo; X = C, N; T = O, OH; *n* = 1–3) in H_2_SO_4_ electrolyte [82]. The predicted capacitance performance of Ti_3_C_2_T_x_, Mo _2_CT_x_, and V_2_CT_x_ [82,83] were similar to the experimentally measured capacitances 235, 245, 90, and 380 F g^−1^, respectively [77,79,82].

**Figure 9:**
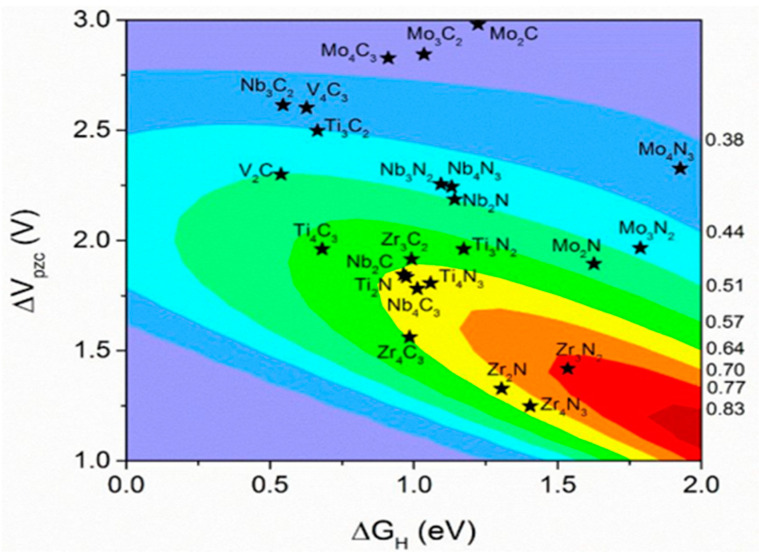
Color map of the relationship between the charge storage per formula unit against the shift in the V_PZC_ and H_2_ adsorption free energy (ΔG_H_). The applied potential ranged from −1 to 1 V vs. standard Hydrogen Electrode (SHE). Reproduced with permission from [82]. Copyright ACS, 2019.

Evaluating the descriptors for the capacitance trends, we find that more positive hydrogen adsorption free energy (weak binding to H) and smaller change of the potential at the point of zero charge after H binding lead to higher capacitance. Interestingly, the pseudocapacitive performance of nitride MXenes electrodes outperformed carbide MXenes. Mainly, Ti_2_NT_x_ is expected to possess a high gravimetric capacitance under any applied voltage in H_2_SO_4_, owing to the low atomic weight and favorable redox chemistry of Ti. Meanwhile, Zr_n+1_N_n_T_x_ is anticipated to possess the best areal capacitive performance [82]. The higher capacitance performance is attributed to the higher adsorption free energy and lower change of the potential at the point of zero charge after H binding [82]. The relationship between the charge storage of nitride and carbide MXenes against the shift in the point-of-zero-charge (V_PZC_) and H_2_ adsorption free energy (ΔG_H_) displayed that the large ΔG*H* and the low Δpzc lead to higher charge storage per unit of formula (Figure 9) [82]. Thereby, Zr-based nitride MXenes (Zr_2_N, Zr_3_N_2,_ and Zr_4_N_3_) reveal the highest charge storage under an applied potential range from −1 to 1 V vs. standard Hydrogen Electrode (SHE) [82].

Although the tremendous progress in the capacitance performance MXenes, some remaining gaps exist among the theoretical calculations and experiments, such as inaccurate consideration of the multilayered structures of MXenes along with ignoring the F-rich MXenes surface [84,85]. 

Increasing the specific surface areas (SSA) and the active redox sites of MXenes can enhance their capacitance performance. Using these strategies, the capacitances of macroporous Ti_3_C_2_T_x_ and Ti_3_C_2_T_x_ hydrogels reached 210 and 380 F g^−1^, respectively, owing to their abundance of active sites resulted from the high SSA [84,109]. Moreover, macroporous Ti_3_C_2_T_x_ shows capacitances of 310, 210, and 100 F g^−1^ at scan rates of 0.01, 10, and 40 V s^−1^, respectively [78]. This indicated the direct correlation between the current density peak (*i*) current and scan rate (*v*), which can be an indicator for the inherent charge storage kinetics as can be calculated using this equation *i* = *av^b^*, where *a* and *b* are constants. Electrodes of supercapacitors usually possess a linear relationship between *v* and *i,* i.e., (i~v). 

To this end, macroporous Ti_3_C_2_T_x_ in H_2_SO_4_ electrolyte showed a pseudocapacitive behavior as found in the linear dependence of log *i* vs. log *v,* i.e., *b ≈* 1 [78].

Another factor for improvement of the capacitance performance of MXenes is the selection of appropriate electrolyte, due to the difference in the ionic conductivity, operation voltage, the temperature of different electrolytes that subsequently tailor the capacitance performance. Thereby, aqueous electrolytes with their outstanding ionic conductivity are preferred compared to organic or ionic liquids electrolytes, although the ionic liquids electrolytes have the largest potential window and feasible for high working temperatures. This finding was achieved in the superior capacitance of Ti_3_C_2_T_x_ (325 Fg^−1^) in H_2_SO_4_ [81], compared to (70 F g^−1^) in ionic liquid electrolytes [110] and (32 F g^−1^) in organic electrolytes [104]. The capacitance performance of Ti_3_C_2_T_x_ in propylene carbonate (PC) organic electrolyte with higher ionic conductivity was higher than that in acetonitrile and dimethyl sulfoxide (DMSO), with lower ionic conductivity [111]. The preparation method of MXenes is also an essential factor for boosting the mechanical properties and electrochemical or capacitance performance. Investigation of the mechanical evolution during the intercalation/deintercalation of MXenes revealed that Li-ion intercalation increases the out-of-plane stiffness (elastic properties) in aqueous electrolytes [59]. This is achieved by proposing a theoretical correlation among the cation content and the out-of-plane elastic properties during electrochemical reactions. 

Although MXenes were reported to be promising for energy storage applications, their restacking issue, low intrinsic electronic and ionic conductivity, and low specific capacity hinder their use in the practical applications [24]. Besides, the underlying mechanism of the use of MXenes for supercapacitors still needs to be clarified, and that requires further in-depth theoretical and experimental investigations. Furthermore, due to the remarkable influence of the electrolytes on the MXene supercapacitors, more studies are needed for electrolytes optimization. 

## 4. Mechanical of Self-Standing MXenes vs. Hybrid MXenes

**Figure 10:**
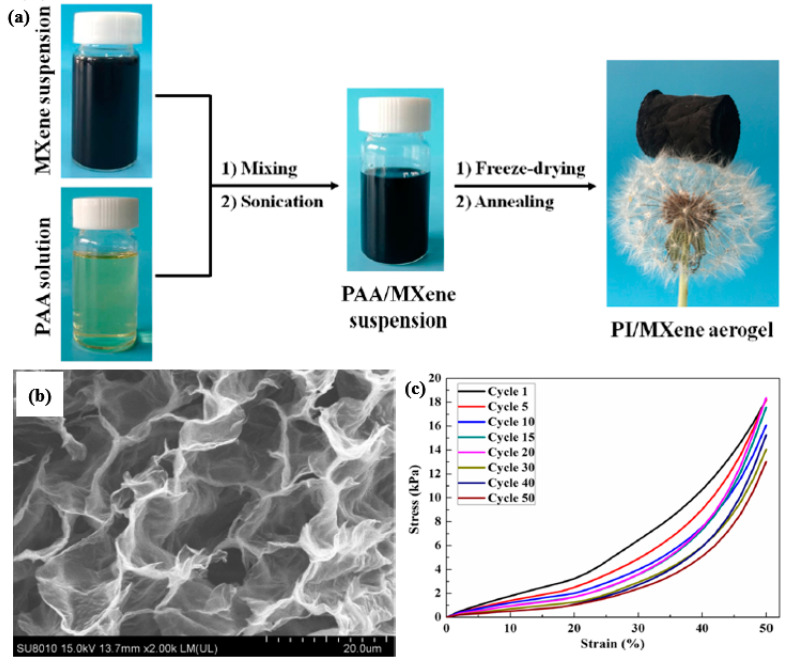
(**a**) Preparation route of PI/Ti_3_C_2_T_x_ aerogels (**b**) Internal structure of PI/Ti_3_C_2_T_x_-3 aerogels (**c**) Stress−strain curves for 50 repeated compression cycles on the PI/MXene-3 aerogel at 50% strain. Adapted and reproduced with permission from [113]. Copyright ACS, 2019.

MXenes, especially Ti_3_C_2_T_x,_ was found to be a promising candidate for enhancing the mechanical properties of various polymers, metals, and carbon materials. This is owing to the multilayered 2D structure and outstanding Young’s modulus of Ti_3_C_2_T_x_ monolayer (0.33 ± 0.03 TPa), measured via the nanoindentation experiments [2]. For instance, the mechanical properties of polyvinyl alcohol (PVA) nanofibers were significantly enhanced via using Ti_3_C_2_T_x_ and cellulose nanocrystals (CNC) fillers (denoted as PVA/CNC/Ti_3_C_2_T_x_) compared to pristine PVA [112]. Notably, PVA nanofibers containing 0.07 wt.% of both CNC and Ti_3_C_2_T_x_ displayed more than 100% enhancement of the storage modulus relative to PVA nanofibers. In comparison, PVA nanofibers with 3 wt.% nanocellulose (PVA/CNC) revealed a 74% increase in storage modulus of PVA at 25 °C [112]. Additionally, the elastic modulus of PVA/CNC/Ti_3_C_2_T_x_ nanofibers (855 MPa) was 2.1 times higher than that of PVA nanofibers (392 MPa). The Young’s modulus of PVA/CNC/Ti_3_C_2_T_x_ nanofibers (293 ± 59 MPa.) was higher than that of PVA/CNC (241 ± 51 MPa), PVA/Ti_3_C_2_T_x_ (283 ± 60 MPa)_,_ and PVA nanofibers (221 ± 51 MPa) [112]. Likewise, polyimide/Ti_3_C_2_T_x_ aerogel prepared via the freeze-drying of and annealing to form a robust, lightweight, and hydrophobic aerogel (Figure 10a) with three-dimensional “house of cards” structure (Figure 10b) [113]. The compressive strength at 80% strain and Young’s modulus of elasticity for PI/Ti_3_C_2_T_x_ aerogel increased significantly with decreasing the Ti_3_C_2_T_x_ concentration. This is owing to greater porosity and lower density of PI/MXene aerogels with the increase of the Ti_3_C_2_T_x_ amount [113]. Interestingly, the elastic properties, PI/MXene-3 with a ratio of 5.2:1, respectively, showed impressive stress−strain repeatability after 50 cycles of compression-release (Figure 10c), attributed to the strong interactions between PI chains and Ti_3_C_2_T_x_ nanosheets in the hybrid aerogel [113]. Silver nanowires, combined with Ti_3_C_2_T_x_ (AgNWs-Ti_3_C_2_T_x_) transparent conductive electrode, displayed a higher conductivity, chemical stability, and mechanical stability than that of pristine AgNW electrode [114].

**Figure 11:**
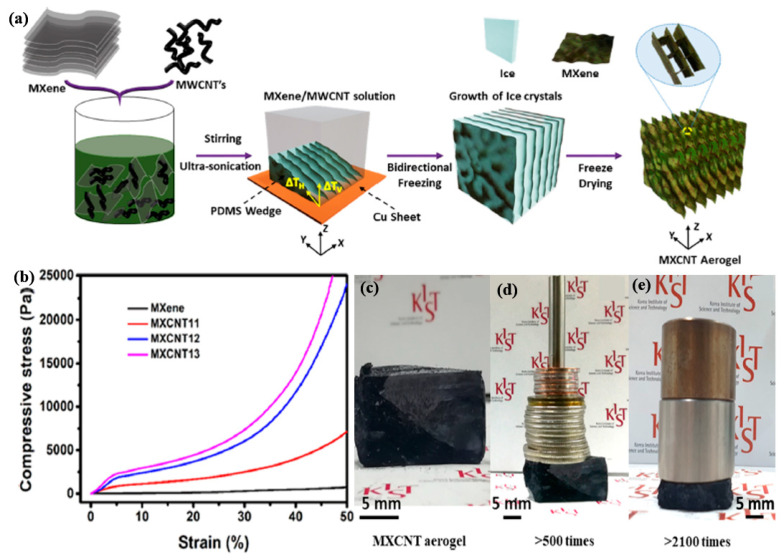
(**a**) Preparation of MXene/CNT hybrid aerogels through bidirectional freezing approach (**b**) Stress-strain curves of pristine MXene aerogels and MXene/CNT hybrid aerogels samples (**c**–**e**) MXene/CNT hybrid aerogels supporting more than >500 and >2100 times of its weight with no obvious collapsing. Adapted and reproduced with permission from [115]. Copyright ACS, 2019.

Ti_3_C_2_T_x_/carbon nanotube (CNT) 3D porous aerogel (denoted as MXCNT) was synthesized using the bidirectional freezing approach (Figure 11a) [115]. Figure 11b displays the compressive stress-strain curves for Ti_3_C_2_T_x_ and MXCNT aerogels measured under compression at a displacement rate of 1 mm/min up to 50% strain. The compressive strength of MXCNT was substantially higher than that of Ti_3_C_2_T_x_ Figure 11b. Also, the compressive strength of MXCNT increased with increasing CNT concentration to reach the maximum value of 25,000 Pa using a ratio of 1/3 of Ti_3_C_2_T_x_/CNT, respectively. This is originated from the uniform distribution of Ti_3_C_2_T_x_ multilayered sheets with CNT in the direction of the compressive force resulting in a uniform aerogel, as shown in (Figure 11c). Interestingly, the as-formed MXCNT aerogel can afford more than 500 times (Figure 11d) and more than 2100 times (Figure 11d) of its weight without collapsing along with recovery of 12.1 % strain after eliminating the applied load. The significant enhancement in the compressive strength of MXCNT is ascribed to the ordered porous framework supported by vertical pillars, that warrants the cell walls deformation on compression rather than sliding between the walls [115]. The MXCNT aerogel is highly promising for electromagnetic interference (EMI) shielding applications. 

## 5. Summary and Perspectives

In summary, this review emphasized the recent advances in the mechanical properties of self-standing MXenes, including elastic properties, bending rigidity, and adhesion and sliding resistance from the experimental and theoretical views. This is, besides, to compare the mechanical properties of self-standing MXenes with hybrid MXenes along with their utilization as supercapacitors. Both experimental and theoretical calculations implied the significant effect of shape (i.e., layer thickness, interlayer spacing, dimensional, and porosity), preparation method, type (i.e., carbides or nitrides), composition (i.e., mono-/binary/multi-metals, doping, defects, and decoration with nanoparticles or single atoms), and functional groups (O, OH, and F) on enhancement the mechanical properties of MXenes. These features endowed the mechanical properties of MXenes are found to be closer to various 2D materials such as graphene, molybdenum disulfide, and boronitrene.

Despite the significant progress achieved in the rational design of self-standing MXenes, their mechanical properties are frequently investigated theoretically rather than experimentally. Additionally, the preparation approaches of MXenes entail multiple complicated steps, hazard chemicals, and without precise monitoring, shape, composition, and surface/bulk functionalities. However, the theoretical calculations predicted the synthesis of dozens of MXenes with outstanding mechanical merits coupled with electrical conductivity, high surface area, and ion adsorption/storage properties, which leaves extensive gates for the utilization of MXenes in various applications such as flexible devices, energy production/storage devices, and sensors. To this end, the capacitance performances of MXenes were enhanced significantly via their integration with conductive polymers, carbon-based materials (i.e., graphene, carbon nanotubes), and doping or functionalization metals (i.e., transition metals, noble metals, non-metals traces, semiconductors). Thereby, the mechanical properties of self-standing MXens and their mechanism should be highlighted experimentally rather than through theoretically. Also, the combination between MXenes and other carbon-based materials and novel metallic nano architectonics can lead to impressive properties and applications [116,117,118,119]. Thus, the presented review can provide a guided roadmap for the scientists to design novel MXenes for the coming generations of energy conversion and storage devices as well as smart sensors.

## Figures and Tables

**Table 1:** The mechanical properties of MXenes with different compositions.

Materials	Morphology	Preparation Method (Experimentally/Theoretically)	Measurements	Elastic Constants c_11_ [GPa]	Young’s Modulus E [GPa]	Strains along Uniaxial x (ε_x_)	Strengths along Uniaxial x (σ_x_) [GPa]	Ref.
Ti_3_C_2_H_2_	2D unit cell	Theoretical calculations	VASP/PBE	419	392	-	-	[27]
Zr_3_C_2_O_2_	2D hexagonal lattice	Etching Al layers in Zr_3_AlC_5_	DFT	392.9	-	-	-	[28]
Ti_2_C	2D sheets	Theoretical calculations	VASP	609	-	-	-	[29]
Ti_2_CO_2_	2D sheets	Theoretical calculations	Nanoindentation process	-	983	-	-	[30]
Ta_2_C	2D sheets	Theoretical calculations	CASTEP/Wu-Cohen	788	-	-	-	[31]
Ti_2_C	2D sheets	Theoretical calculations	MD	-	597	-	-	[32]
Mo_2_C	2D sheets	Chemical vapor deposition	VASP	-	312	-	-	[33]
Ti_2_CO_2_	2D sheets	Etching Al layers in Ti_2_AlC_2_	VASP/PBE	745	570	0.28	56	[34]
W_2_C	2D sheets	Theoretical calculations	VASP/PBE	781.9	-	0.16	65.6	[35]
**Materials**	**Morphology**	**Preparation method**	**Measurements**	**c_11_** **(N/m)**	**E** **(N/m)**	**ε_x_**	**σ_x_** **(N/m)**	**Ref.**
Ti_3_C_2_O_2_	2D unit cell	Theoretical calculations	VASP/PBE	379	347	-	-	[27]
W_2_HfC_2_O_2_	2D unit cell	Theoretical Calculations	VASP/PBE	-	-	-	47.3	[34]
Mo_2_CO_2_	hexagonal unit cell	Theoretical calculations	VASP/PBE	361	302	-	-	[35]
Ti_2_CO_2_	2D sheets	Theoretical Calculations	DFT	-	241	0.24	30.7	[36]

**Table 2:** Freestanding Ti_3_C_2_T_x_ MXenes prepared by various approaches as efficient supercapacitors compared to some Ti_3_C_2_T_x_ composites as a function of preparation method. Abbreviations: PPy = polypyrrole, rGO = reduced graphene oxide, CNT = carbon nanotubes and EG = electrochemically exfoliated graphene.

MAX Phase MXene-Hybrid	Material Composition	Synthesis/Characterization Methods	Morphology	Performance	Ref.
Ti_3_AlC_2_	Ti_3_C_2_T_x_	HF etching/TEM, SEM, CA, XRD, EIS	Nanosheets	517 F/g at 1 A/g	[16]
Ti_3_AlC_2_	Ti_3_C_2_T_x_	HF etching/EIS, XRD	Paper	340 F/cm^3^ at 1 A/g	[76]
Ti_3_AlC_2_	Ti_3_C_2_T_x_-P	HCl–LiF/SEM, TEM, FTIR	Paper	416 F/g at 5 mV/s	[84]
Ti_3_AlC_2_	Ti_3_C_2_T_x_	HCl–LiF/XRD, TEM, SEM	Nanosheets	900 F/cm^3^ at 2 mV/s	[77]
Ti_3_AlC_2_	Ti_3_C_2_T_x_-EG	HF etching/XRD, TEM, SEM	Nanosheets	33 F/cm^2^ at 2 mV/s	[86]
Ti_3_AlC_2_	Ti_3_C_2_T_x_	HF etching/NA	Film	528 F/cm^3^ at 2 mV/s	[87]
Ti_3_AlC_2_	Ti_3_C_2_T_x_	NH_4_F-hydrothermal/XRD, SEM, Raman, XPS	Nanosheets	141 F/g at 2 A/g	[88]
Ti_3_AlC_2_	Ti_3_C_2_T_x_-rGO	HCl–LiF/XRD, SEM	Nanosheets	8.6 mWh/cm^3^ at 0.2 W/cm^3^	[89]
Ti_3_AlC_2_	Ti_3_C_2_T_x_-CNT	HCl–LiF/XRD, SEM, TEM	Nanosheets	314 F/cm^3^ at 1.7 mg/cm^2^	[90]
Ti_3_AlC_2_	Ti_3_C_2_T_x_	HF etching/NA	Nanosheets	2.8 mWh/cm^3^ at 0.225 W/cm^3^	[91]
Ti_3_AlC_2_	BiOCl-Ti_3_C_2_T_x_	HF etching/XRD, SEM, TEM, XPS	Nanosheets	397 F/cm^3^ at 1 A/g	[92]
Ti_3_AlCN	Ti_3_C_2_T_x_	HCl–LiF/TEM, AFM, SEM	Nanosheets	61 mF/cm^2^ at 5 μA/cm^2^	[93]

**Table 3:** Freestanding Ti_3_C_2_T_x_ and Ti_2_CT_x_ MXenes supercapacitors compared to some Ti_3_C_2_T_x_ composites as a function of electrolyte and scan rate/current density. Abbreviations: PPy = polypyrrole, rGO = reduced graphene oxide, CNT = carbon nanotubes, PVA = polyvinyl alcohol, and SWCNT = single-walled carbon nanotubes.

Freestanding Ti_3_C_2_T_x_ and Ti_2_CT_x_ MXenes						
Electrode	Electrolyte	Scan Rate/Current Density	Initial Capacitance (IC)	Cycle Number (CN)	Capacity After Cycles (AC)	Ref.
Ti_2_CT_x_	30 wt % KOH	10 A g^−1^	51 F g ^−^^1^	6000	93%	[74]
Ti_3_C_2_T_x_	1 M KOH	1 A g ^−^^1^	350 F cm^−3^	10,000	∼94%	[76]
Ti_3_C_2_T_x_	1 M H_2_SO_4_	5 A g ^−^^1^	415 F cm^−3^	10,000	∼100%	[81]
Ti_3_C_2_T_x_	1 M H_2_SO_4_	10 A g ^−^^1^	900 F cm^−3^	10,000	∼100%	[77]
Ti_3_C_2_T_x_	1 M H_2_SO_4_	10 A g ^−^^1^	499 F g ^−^^1^	10,000	∼100%	[94]
Ti_3_C_2_T_x_	6 M KOH	5 A g ^−^^1^	118 F g^−1^	5000	∼100%	[95]
Ti_3_C_2_T_x_	1 M H_2_SO_4_	5 A g ^−^^1^	215 F g^−1^	10,000	∼100%	[96]
Ti_3_C_2_T_x_	1 M H_2_SO_4_	5 A g ^−^^1^	892 F g^−1^	10,000	∼100%	[97]
N-Ti_3_C_2_T_x_	1 M H_2_SO_4_	50 mv s ^−1^	192 F g ^−^^1^	10,000	92%	[98]
Ti_3_C_2_T_x_/paper	1 M H_2_SO_4_	2 mA cm^−2^	25 mF cm^−2^	10,000	92%	[99]
Ti_3_C_2_T_x_/3D porous layered double hydroxide	6 M KOH	1 A g^−1^	1061 F g^−1^	4000	70%	[100]
400-KOH-Ti_3_C_2_T_x_	1 M H_2_SO_4_	1 A g^−1^	517 F g^−1^	10,000	>99%	[101]
**Ti_3_C_2_T_x_ MXenes Hybrid Composites**						
**Electrode**	**Electrolyte**	**Scan Rate/Current Density**	**Initial Capacitance (IC)**	**Cycle Number (CN)**	**Capacity After Cycles (AC)**	**Ref.**
Ti_3_C_2_T_x_/PVA	1 M KOH	5 A g ^−^^1^	∼370 F cm^−3^	10,000	∼85%	[16]
PPy/Ti_3_C_2_T_x_	1 M H_2_SO_4_	100 mV s^−1^	∼250 F g^−1^	25,000	92%	[84]
Ti_3_C_2_T_x_/SWCNT	1 M MgSO_4_	5 A g ^−^^1^	345 F cm^−3^	10,000	∼100%	[102]
Ti_3_C_2_T_x_/rGO	3 M H_2_SO_4_	100 mV s^−1^	777 F cm^−3^	20,000	∼100%	[103]
Ti_3_C_2_T_x_/CNT	1 M EMITFSI	1 A g^−1^	∼80 F g^−1^	1000	∼90%	[104]
Ti_3_C_2_T_x_/CNT	6 M KOH	10 mv s^−1^	∼384 F g^−1^	10,000	∼100%	[105]
TiO_2_/Ti_3_C_2_T_x_	6 M KOH	5 mV s^−1^	143 F g^−1^	3000	∼96%	[106]
MnO_x_/Ti_3_C_2_T_x_	1 M Li_2_SO _4_	2 mV s^−1^	602 F cm^−3^	10,000	89.8%	[107]
PPy/Ti_3_C_2_T_x_	0.5 M H_2_SO_4_	1 mA cm^−2^	406 F cm^−3^	20,000	∼96%	[108]
